# Childhood Trauma Is Associated with the Spirituality of Non-Religious Respondents

**DOI:** 10.3390/ijerph17041268

**Published:** 2020-02-17

**Authors:** Alice Kosarkova, Klara Malinakova, Zuzana Koncalova, Peter Tavel, Jitse P. van Dijk

**Affiliations:** 1 Olomouc University Social Health Institute, Palacky University Olomouc, 771 11 Olomouc, Czech Republic; klara.malinakova@oushi.upol.cz (K.M.); peter.tavel@oushi.upol.cz (P.T.); j.p.van.dijk@umcg.nl (J.P.v.D.); 2 Department of Community and Occupational Medicine, University Medical Center Groningen, University of Groningen, 9713 AV Groningen, The Netherlands; 3 Graduate School Kosice Institute for Society and Health, P.J. Safarik University in Kosice, 040 11 Kosice, Slovak Republic

**Keywords:** childhood trauma, abuse, neglect, spirituality, religiosity, conversion

## Abstract

Childhood trauma experience (CT) is negatively associated with many aspects of adult life. Religiosity/spirituality (R/S) are often studied as positive coping strategies and could help in the therapeutic process. Evidence on this is lacking for a non-religious environment. The aim of this study was to assess the associations of different types of CT with R/S in the secular conditions of the Czech Republic. A nationally representative sample (*n* = 1800, mean age = 46.4, SD = 17.4; 48.7% male) of adults participated in the survey. We measured childhood trauma, spirituality, religiosity and conversion experience. We found that four kinds of CT were associated with increased levels of spirituality, with odds ratios (OR) ranging from 1.17 (95% confidence interval 1.03–1.34) to 1.31 (1.18–1.46). Non-religious respondents were more likely to report associations of CT with spirituality. After measuring for different combinations of R/S, each CT was associated with increased chances of being “spiritual but non-religious”, with OR from 1.55 (1.17–2.06) to 2.10 (1.63–2.70). Moreover, converts were more likely to report emotional abuse OR = 1.46 (1.17–1.82) or emotional neglect with OR = 1.42 (1.11–1.82). Our findings show CT is associated with higher levels of spirituality in non-religious respondents. Addressing spiritual needs may contribute to the effectiveness of psychotherapeutic treatment of the victims.

## 1. Introduction

Religiosity and spirituality (R/S) represent for many people important dimensions of their everyday lives, and they are also increasingly associated with human health. Spirituality is nowadays seen as an individual’s contentedness towards a Higher Power, a search for harmony, a sense of the meaning of life and spiritual well-being [1,2], whereas religiosity can be described in terms of church attendance, institutional beliefs and rituals and theology prescribed by a particular institution [3]. Studies mostly show a positive impact of both R/S on healthy attitudes and behavior [4,5]. Moreover, associations can also be found between R/S and self-rated health and life satisfaction as well as both physical and mental health [6].

Specifically, in the area of mental health spirituality is connected with a lower occurrence of anxiety and depressive symptoms [7,8] and represents a strong coping strategy for dealing with serious illnesses or difficult life situations [9,10,11]. However, research also shows negative associations of so-called religious and spiritual struggles with health [12,13]. Furthermore, it shows the impact of negative religious coping on worse quality of life, lower life satisfaction [14], worse physical functioning [15] and a decline in health [12]. These findings highlight the need for understanding under what conditions people tend to use either positive or negative religious coping.

Research shows that R/S tends to be shaped by family arrangement, upbringing and peer attachment [16]. It may also be partially influenced by personality traits, though no causality has thus far been proved [17]. Nevertheless, the experience of childhood trauma seems to be a strong factor associated with R/S development. R/S may be influenced by a traumatic event in a positive but also in a negative way [18]. In some cases, trauma resulted in post-traumatic growth [19] or led to the use of R/S as a coping strategy as a means of recovering through the use of prayer, Holy Scripture, dialogue with a middle-man or faith in a Higher Power’s justice [20]. In a negative sense, some victims of abuse deny faith in a Higher Power or stop their religious attendance [21]. In these victims, a lower score in different spirituality dimensions, such as a reason for living, perceiving one’s productivity or feeling peaceful after surviving a trauma, is also observed [22]. Research shows that depressive symptoms are more likely to develop among those who have experienced abuse and that their R/S decreases after a traumatic experience [23].

The Czech Republic, as one of the post-communist countries, is characterized by a high degree of secularization, as most people do not report any religion affiliation or regular church attendance [24]. Therefore, the aim of this study is to assess the associations of different types of childhood trauma with R/S among Czech adults in a secular environment, taking into account different combinations of respondents’ spirituality and religiosity status.

## 2. Materials and Methods 

### 2.1. Participants and Procedure

We used a national sample of the Czech population aged fifteen years and older. We conducted a two-step process. The first step was a pilot procedure realized among 206 participants. This process led to the final version of the survey. The second step was the recruitment of another 2184 participants chosen randomly by a quota sampling and asked to participate in a study on the issues of health, life experiences and attitudes and lifestyle. Quota sampling is a sampling technique often used in research to imitate the known characteristics of the population in the sample, allowing relationships between subgroups to be observed. In this case the criteria that allowed the construction of a representative sample corresponding to the adult Czech population (aged 15 years and over) with regards to gender, age, education and religious affiliation were used. Of these participants, 384 (17.6%) refused to conduct the survey due to lack of time (39.2%), lack of interest in the research or distrust in general (24.0%) or the length and difficulty of questionnaire questions (11.2%). 

Professionally trained administrators collected data in September and October 2016 using standardized face-to-face interviews. Participation in the survey was anonymous and voluntary. The final sample consisted of 1800 respondents. The study design was approved by the Ethics Committee of the Olomouc University Social Health Institute, Palacky University Olomouc (No. 2016/3).

### 2.2. Measures

All instruments were available in the Czech language.

Childhood trauma was assessed using the Childhood Trauma Questionnaire (CTQ) [25]. The CTQ is a standardized inventory consisting of 28 items, which was developed to assess the importance of five types of abuse and maltreatment experienced in childhood or adolescence. The CTQ corresponds to five subscales: Emotional Abuse, Physical Abuse, Sexual Abuse, Emotional Neglect and Physical Neglect. Each of the subscales consists of five items rated on a 5-point Likert-type scale within the range from “never” (1) to “very often” (5), resulting in scores from 5 to 25 for each subscale. The possibility of quantifying the degree of maltreatment and abuse makes this instrument unique [25]. We used the CTQ validated for Czech conditions [26], which was introduced by the statement “The following questions are related to some of your childhood or adolescent experiences” in order to be sure that the trauma occurred in childhood/adolescence. Cronbach’s alpha for the CTQ subscales in our sample ranged from 0.62 to 0.89. 

Religiosity was measured with the question: “At present, would you call yourself a believer?” with possible answers: “yes, I am a member of a church or religious society”; “yes, but I am not a member of a church or religious society”; “no”; “no, I am a convinced atheist”.

Spirituality was measured using the Daily Spiritual Experience Scale (DSES) [27]. The scale measures the frequency of ordinary experiences of connection with transcendence in everyday life. In the present study an adapted 15-item version of the scale [24] was used. Each item was evaluated on a six-degree Likert scale graded according to the intensity of experiencing the observed phenomena, ranging from “many times a day (1) to “never” (6). A higher intensity of experience corresponds to higher levels of spiritual experience. For the analysis we used the total score as a continuous variable. Cronbach’s alpha was 0.96.

Religiosity and spirituality were further combined into four groups—“religious/spiritual”, “non-religious/spiritual”, “non-spiritual/religious” and “non-religious/non-spiritual”—in order to distinguish between spiritual experience and religious affiliation and to assess their interaction. 

Conversion experience was assessed with the question “Have you ever experienced something that could be called a religious conversion (acceptance or change of denomination)?” with possible answers: yes; no. The term denomination means acceptance of a new religion, which is broader than just a change within the same religion. Those answering in a positive way were further considered as converts. 

The background characteristics gender, age, education level, marital status and economic activity were obtained by means of a questionnaire. Education level was obtained by marking the highest educational level achieved from elementary, secondary vocational, secondary graduation and college education. The marital status question offered the possibility of choosing from living alone statuses or from living with a partner status. Economic activity assessed employment, unemployment, self-employment, being a student or retiree. 

### 2.3. Statistical Analyses

In the first step, the background characteristics of the sample and the observed categorical variables were described, comparing the groups with four different R/S combinations. We then assessed the associations of different childhood trauma, standardized to z-scores, with spirituality using a binary logistic regression, first crude and consequently adjusted for gender, age, education and marital status. In the next step, the associations of different kinds of childhood trauma, assessed as a continuous variable standardized to z-scores, with non-religious/spiritual groups, religious/spiritual and religious/non-spiritual groups were analysed using a multinominal logistic regression. The models were assessed crude and adjusted for gender, age and educational level. Finally, the analyses were also repeated for the association of childhood trauma and experience of conversion. All analyses were performed using the statistical software package IBM SPSS version 25 (IBM Corp., Armonk, NY, USA).

## 3. Results

### 3.1. Description of the Population

The background characteristics of the sample are presented in Table 1. The sample is representative of the Czech population aged 15 years and over (mean age 46.4, SD = 17.4; 95% CI = 45.60–47.21; 48.7% men). Of the whole sample, 349 respondents (19.4%) described themselves as religious but non-spiritual, and 182 respondents (10.1%) as both religious and spiritual. Furthermore, 60 participants reported having had a conversion experience. 

### 3.2. Adult Spirituality

As the first step, we assessed the associations of religiosity with different children trauma experiences; however, the results were not significant. Nevertheless, assessing spirituality (see Table 2) showed significant positive results, with ORs ranging from 1.17 (95% confidence interval 1.03–1.34) to 1.31 (1.18–1.46) for the adjusted group. Furthermore, when respondents were divided according to their religiosity the ORs ranged from 1.55 (1.17–2.06) to 2.10 (1.63–2.70) for the non-religious adjusted group. For the whole sample, each kind of traumatic experience was associated with an increased level of adult spirituality, with the exception of emotional neglect. When the complete sample was split into religious and non-religious respondents, the associations in the religious group were statistically insignificant for any type of trauma, while there was an increase in the odds of spirituality for the non-religious group. Moreover, emotional neglect also showed a significant association with spirituality (*p* < 0.01) when assessed separately in the non-religious respondents.

### 3.3. Religiosity/Spirituality

The results of multinominal logistic regression are shown in Table 3. Different combinations of respondents’ R/S revealed that all kinds of childhood trauma are significantly (*p* < 0.01 or *p* < 0.001) associated with increased chances of being non-religious/spiritual, where the highest chances were found for physical neglect with OR = 2.10 (1.63–2.70) in the adjusted model. In contrast, the associations of childhood trauma with other R/S combinations were not significant.

### 3.4. Experience of Conversion

Table 4 depicts the associations of childhood trauma with a conversion experience. Respondents who reported emotional abuse or emotional neglect were more likely to have had a conversion experience (*p* < 0.01), whereas the other kinds associations with the other kinds of trauma were not significant.

## 4. Discussion

The aim of this study was to assess the associations of childhood trauma experience with adult R/S in a secular environment. The results indicated that individuals who have an experience of childhood traumatic event(s) are more likely to achieve a higher spirituality score. However, the differences between the groups with different combinations of R/S suggest that childhood trauma experience increases the chances of being spiritual only among non-religious individuals, as the association among the others were not significant. Furthermore, we found that the respondents with an experience of emotional abuse or emotional neglect were more likely to report having had a conversion experience. 

We found that all kinds of childhood trauma, except emotional neglect, were associated with an increase in adult spirituality throughout the complete sample. However, emotional neglect was indeed associated with an increased level of spirituality, but only in non-religious respondents. Our findings are consistent with previous research describing the effect of childhood trauma experience on adult spirituality [28,29,30]. An explanation may be that spirituality helps in the effort to incorporate the traumatic event into life and can offer support in the recovery process [31]. Similarly, spirituality may offer an opportunity for personal post-traumatic growth, which can be characterized by a change of self-perception, relationship enhancement or greater resilience, and a change of life philosophy, such as a new appreciation of life or spiritual beliefs [32]. Several studies have shown spirituality to have a positive effect on victims’ recovery process after trauma and to be connected to positive changes in trauma survivors’ lives [30,32]. 

Our second finding—that religious respondents reported no increase in their adult spirituality following a childhood trauma experience—is in line with other authors who report that for religious people it may be more difficult to reappraise the meaning of trauma. Such trauma is connected to their core beliefs and may cause a shift from a benevolent and protective Higher Power to a Higher Power that is distant, allows suffering to occur and/or has abandoned them [30,33]. Thus, religious respondents may be even more likely to experience a decrease in spirituality [18]. Moreover, it is possible that religious respondents already had some level of spirituality [3] before the trauma occurred and they keep it regardless of the trauma. Thus, in this situation we would not observe any significant increase in spirituality. However, there is a partial discrepancy with previous studies, which found a negative relationship between trauma and later R/S [22,29] and in which victims associated their trauma experience with the Higher power’s punishment and the desire to keep the Higher Power at a distance [30,34]. 

Furthermore, having assessed various R/S combination groups, we found that all kinds of childhood trauma are associated with an increased level of being spiritual but non-religious. This may help to understand the mixed findings on associations between trauma and R/S in various studies (see [35] for review). A possible explanation may be that only some studies assessed the combination of both religiosity and spirituality with trauma (e.g., [36,37]), while the majority of studies assessed associations of trauma either with religiosity (e.g., [18]) or spirituality (e.g., [29,38]) or kept a distinction between religiosity and spirituality as separate constructs [39]. However, R/S is a multifaceted construct that includes attitudes, behavior and beliefs [40], and various measures are used to access any part of this construct and spiritual change particularly [18] and thus may lead to different results. Moreover, we consider our findings of an increased level of being spiritual but not religious to be a consequence of an effort to incorporate trauma into one’s life and reappraise its meaning [41]. Non-religious people in their meaning-making after trauma may search for something outside the material word to help them reappraise their existential despair and find a new global meaning after their basic trust was violated. They may be disappointed by the material world and turn to the sacred [42] and to individual spirituality independently of any organized forms. In addition, we can assume that in the secular Czech environment the search for the sacred and the meaning of life after a traumatic experience is not connected with religiosity due to the specific historical and cultural background. There are a lot of prejudices against churches and organized forms of religion in Czech society [43] as well as difficulties in the relationship between the non-religious and members of a church [44] when dealing with a traumatic event. 

Finally, our findings that emotional abuse and emotional neglect are associated with higher chances of a conversion experience further support the connection of childhood trauma with adult R/S, i.e., an individual’s desire for a connection to a Higher Power, either in the form of an organized system (religion) or in the form of non-institutional identification with the sacred. In order to cope with trauma, victims use various coping strategies, and R/S is a well-documented strategy that may help a person understand and deal with stressors [20,45]. We suggest that even for non-believers, identification with the sacred can become a source of self-significance [46], and R/S may alter their understanding of suffering not fulfilled by the material world. Moreover, the conversion experience could stem from emotional and relationship needs [47], which may lead to the search for security and a safe haven. Thus, our results are in line with the compensational hypothesis [48] that connects insecure attachment with spiritual development. Victims of emotional abuse or neglect may not have developed secure relationships [38] and may involve God in their lives as a substitute figure and source of emotional security [49].

### 4.1. Strengths and Limitations

One of the main strengths of our study is the large representative sample and its realization in a mostly secular environment, where no strong affiliation to any religion is present. Therefore, the results are more likely to be independent of any religious specificity, such as an image of a Higher Power or a typical religious practice. The second strength is the assessment of various types of childhood trauma and abuse in combination with both religious affiliation and spirituality.

There are some limitations in our study, as well. Related to previous research, we consider the fact that we don’t know the exact source of trauma—who or what caused it—as a limitation. Moreover, another limitation is that we have no information about the time between the childhood trauma and the conversion experience or whether the victims received any psychiatric treatment. Furthermore, we don’t know whether any of the 384 respondents who refused to participate in the study had undergone some childhood trauma experience, which could cause selection bias. The next limitation is the cross-sectional design of the study, which does not allow us to make causal inferences. Another limitation may involve information bias, as our data were based on self-reports of respondents, which may be influenced by social desirability. These limitations should be included in a follow-up study in order to achieve a better and more precise understanding of childhood trauma experience on human personality and to know how to help victims cope with traumatic event.

### 4.2. Implications

The results of our study are beneficial for workers in helping professions, such as psychotherapy, psychosomatic medicine, social work or pastoral care. They also contribute to extend the perspective on factors that provide aid those experiencing personal trauma and dealing with trauma. Furthermore, our findings shed light on the way to understand the interconnectedness of aspects affecting the personality of trauma survivors, which could be beneficial in the process of trauma recovery. Moreover, our results stress the idea of internalization of spiritual values, which could support the effectiveness of interventions. Further research should focus on more specific distribution of the respondent groups according their religiosity or spirituality and should also take into account possible sources of trauma and the personality of a perpetrator.

## 5. Conclusions

Our findings show that childhood trauma is associated with R/S in adulthood. However, a significantly higher spirituality following a traumatic experience was observed only among non-religious individuals. Therefore, further research is needed in order to clarify and understand the process underlying the associations between R/S and childhood trauma in order to help victims deal better with traumatic events.

## Figures and Tables

**Table 1:** Description of the study population, total and by religiosity/spirituality (R/S).

	**Total**		**Religious Spiritual**		**Religious Non-Spiritual**		**Non-Religious Spiritual**		**Non-Religious Non-Spiritual**	
	*n*	%	*n*	%	*n*	%	*n*	%	*n*	%
**Gender**										
Male	877	48.7	72	8.2	159	18.1	21	2.4	625	71.3
Female	923	51.3	110	11.9	190	20.6	19	2.1	604	65.4
**Age**										
15–29 years old	410	22.8	19	4.6	67	16.3	11	2.7	313	76.3
30–44 years old	449	24.9	35	7.8	70	15.6	11	2.4	333	74.2
45–59 years old	443	24.6	47	10.6	93	21.0	11	2.5	292	65.9
60–90 years old	498	27.7	81	16.3	119	23.9	7	1.4	291	58.4
**Living Arrangement**										
With husband/wife	921	51.2	110	11.9	195	21.2	19	2.1	597	64.8
With unmarried mate	351	19.5	13	3.7	54	15.4	12	3.4	272	77.5
Alone	353	19.6	42	11.9	74	21.0	6	1.7	231	65.4
With parents/siblings	175	9.7	17	9.7	26	14.9	3	1.7	129	73.7
**Marital Status**										
Single/Divorced/Widow(er)	730	40.6	64	8.8	135	18.5	18	2.5	513	70.3
Married/Partner relationship	1070	59.4	118	11.0	214	20.0	22	2.1	716	66.9
**Highest Education Achieved**										
Elementary school	141	7.8	13	9.2	24	17.0	2	1.4	102	72.3
Secondary vocational school	442	24.6	57	12.9	93	21.0	11	2.5	281	63.6
Secondary school with graduation	854	47.4	74	8.7	160	18.7	17	2.0	603	70.6
College	363	20.2	38	10.5	72	19.8	10	2.8	243	66.9
**Economic Activity**										
Employee	939	52.5	70	7.5	171	18.2	21	2.2	677	72.1
Self-employed	170	9.5	20	11.8	25	14.7	8	4.7	117	68.8
Household ^a^/Unemployed	83	4.6	8	9.6	19	22.9	0	0.0	56	67.5
Student	178	9.9	10	5.6	29	16.3	4	2.2	135	75.8
Disabled/Old-age pensioner	430	23.9	74	17.2	105	24.4	7	1.6	244	56.7
**Religiosity ^b^**										
Believer, church member	170	9.4	107	62.9	63	37.1	0	0	0	0
Believer outside the church	361	20.1	75	20.8	286	79.2	0	0	0	0
Non-believer	1004	55.8	0	0	0	0	32	3.2	972	96.8
Convinced atheist	265	14.7	0	0	0	0	8	3.0	257	97.0
**Total**	1800	100	182	10.1	349	19.4	40	2.2	1229	68.3

Notes: ^a^ including maternity leave, ^b^ independently of church attendance.

**Table 2:** Associations of different childhood trauma experiences with adult spirituality (per standard deviation, scores standardized to z-scores): results of binary logistic regression crude and adjusted for gender, age and education level leading to odds ratios (OR) with 95% confidence intervals (95% CI).

		**Adult Spirituality Crude OR (95% CI)**	**Adult Spirituality Adjusted OR (95% CI)**
Emotional abuse	Complete sample	1.15 (1.01–1.30) *	1.17 (1.03–1.34) *
(per SD) ^a^	NR	1.61 (1.32–1.98) ***	1.64 (1.33–2.02) ***
	R	0.99 (0.83–1.18)	1.01 (0.84–1.21)
Physical abuse	Complete sample	1.29 (1.15–1.44) ***	1.29 (1.16–1.45) ***
(per SD) ^a^	NR	1.72 (1.45–2.03) ***	1.72 (1.45–2.04) ***
	R	1.15 (0.97–1.37)	1.14 (0.96–1.36)
Sexual abuse	Complete sample	1.30 (1.18–1.47) ***	1.31 (1.18–1.46) ***
(per SD) ^a^	NR	1.73 (1.48–2.02) ***	1.73 (1.48–2.03) ***
	R	1.15 (0.97–1.36)	1.16 (0.98–1.38)
Emotional neglect	Complete sample	1.08 (0.93–1.22)	1.04 (0.90–1.19)
(per SD) ^a^	NR	1.54 (1.16–2.04) **	1.55 (1.17–2.06) **
	R	0.95 (0.79–1.13)	0.91 (0.76–1.10)
Physical neglect	Complete sample	1.29 (1.14–1.47) ***	1.24 (0.09–1.41) **
(per SD) ^a^	NR	2.00 (1.57–2.57) ***	2.10 (1.63–2.70) ***
	R	1.16 (0.98–1.39)	1.11 (0.93–1.34)

Notes: * *p* < 0.05, ** *p* < 0.01, *** *p* < 0.001; ^a^ Total number of respondents in each category.

**Table 3:** Associations of different childhood trauma experiences (per standard deviation, scores standardized to z-scores) with R/S combinations: results of multinominal logistic regression crude and adjusted for age, gender and education level leading to odds ratios (OR) with 95% confidence intervals (95% CI).

	Non-Religious/Spiritual		Religious/Spiritual		Religious/Non-Spiritual	
	Crude	Adjusted	Crude	Adjusted	Crude	Adjusted
	OR (95% CI)	OR (95% CI)	OR (95% CI)	OR (95% CI)	OR (95% CI)	OR (95% CI)
Emotional abuse	1.61 (1.32–1.98) ***	1.64 (1.33–2.02) ***	0.99 (0.82–1.18)	1.01 (0.84–1.21)	1.03 (0.92–1.16)	1.05 (0.93–1.19)
Physical abuse	1.72 (1.45–2.03) ***	1.72 (1.45–2.04) ***	1.14 (0.97–1.37)	1.14 (0.96–1.36)	0.99 (0.87–1.13)	0.99 (0.86–1.13)
Sexual abuse	1.73 (1.48–2.02) ***	1.73 (1.48–2.03) ***	1.15 (0.97–1.36)	1.16 (1.00–1.38)	1.00 (0.87–1.15)	1.00 (0.87–1.15)
Emotional neglect	1.54 (1.16–2.03) **	1.55 (1.17–2.06) **	1.95 (0.79–1.13)	0.91 (0.76–1.10)	1.04 (0.92–1.17)	1.01 (0.90–1.14)
Physical neglect	2.00 (1.57–2.58) ***	2.10 (1.63–2.70) ***	1.16 (0.98–1.37)	1.11 (0.93–1.34)	0.98 (0.86–1.10)	0.93 (0.82–1.06)

Notes: ** *p* < 0.01, *** *p* < 0.001; SD = standard deviation.

**Table 4:** Associations of different childhood trauma experiences (per standard deviation, scores standardized to z-scores) with conversion experience: results of binary logistic regression crude and adjusted for age, gender and education level leading to odds ratios (OR) with 95% confidence intervals (95% CI).

	**Conversion Experience**	
	Crude OR (95% CI)	Adjusted OR (95% CI)
Emotional abuse	1.44 (1.16–1.79) **	1.46 (1.17–1.82) **
Physical abuse	1.04 (0.80–1.34)	1.02 (0.78–1.33)
Sexual abuse	0.90 (0.65–1.26)	0.90 (0.64–1.27)
Emotional neglect	1.43 (1.12–1.83) **	1.42 (1.11–1.82) **
Physical neglect	0.99 (0.75–1.30)	0.97 (0.74–1.29)

Notes: ** *p* < 0.01; SD = standard deviation.
